# Effect of Centrifuged Chicken Egg Yolk on the Cryopreservation of Boar Semen

**DOI:** 10.3390/ani15040599

**Published:** 2025-02-19

**Authors:** Fuqiang Chang, Biyu Zhang, Haidong Liu, Henglei Fan, Rui Xie, Jing Li, Qianqian Hu, Chongmei Ruan

**Affiliations:** 1 College of Animal Science, Anhui Science and Technology University, Chuzhou 233100, China; changfuqiang2022@163.com (F.C.); 15255778563@163.com (B.Z.); 15256973237@163.com (H.L.); 15805505713@163.com (H.F.); 17555765516@163.com (R.X.); lij@ahstu.edu.cn (J.L.); 2 Anhui Province Key Laboratory of Animal Nutrition Regulation and Health, Anhui Science and Technology University, Chuzhou 233100, China; 3 Anhui Engineering Research Center of Pork Quality Control and Enhancement, Anhui Science and Technology University, Chuzhou 233100, China

**Keywords:** porcine spermatozoa, chicken yolk, centrifugal processing, cryopreservation, semen quality

## Abstract

Egg yolk, commonly employed as a cryoprotectant in semen cryopreservation, contains large particle matter that can diminish semen quality post thaw and complicate its quality assessment. For this reason, we designed a centrifugal treatment of chicken egg yolk to evaluate its effect on the cryopreservation of porcine semen. The control group (CG) was prepared with a dilution of chicken egg yolk by conventional mixing treatment, and the experimental group (EG) used a dilution of centrifugally treated chicken egg yolk for the ultra-low-temperature cryopreservation of porcine semen. The freezing process was carried out by conventional freezing methods. The spermatozoa were subsequently assessed for various parameters, including motility, acrosome integrity rate, plasma membrane integrity rate, antioxidant indexes, apoptosis rate, and the expression of apoptosis-related genes. The results showed that, post freeze–thawing, the motility, viability, VSL, and VCL of the spermatozoa in the EG were significantly higher than those observed in the CG (*p* < 0.05). Additionally, the acrosome integrity and plasma membrane integrity of the spermatozoa in the EG were significantly enhanced compared to the CG (*p* < 0.05). Furthermore, the EG exhibited significantly lower MDA content and sperm apoptosis rate (*p* < 0.05), while demonstrating significantly higher T-AOC and CAT levels (*p* < 0.05) relative to the CG. In comparison to the CG, the EG exhibited a significant reduction in the gene expression of TNF-a and Bax in the spermatozoa (*p* < 0.05), whereas the expression levels of CAT and Bcl-2 were significantly elevated (*p* < 0.05). In conclusion, the dilution solution formulated through the centrifugal processing of chicken egg yolk demonstrated efficacy in enhancing the quality of porcine spermatozoa following cryopreservation and subsequent thawing.

## Simple Summary

The ultra-low temperature preservation of porcine spermatozoa, utilizing centrifugally processed chicken yolk cryodilution, significantly enhanced motility parameters and acrosome and plasma membrane integrity, elevated antioxidant enzyme levels, and reduced apoptotic gene expression in freeze–thawed porcine spermatozoa.

## 1. Introduction

Semen cryopreservation technology has multiple advantages in animal husbandry production, which can preserve the semen of high-quality breeding males for a long time, reduce the spread of diseases, and decrease the necessity for maintaining large populations of breeding animals, thereby improving economic efficiency. Currently, the methodologies employed for the cryopreservation of porcine semen remain suboptimal. Spermatozoa are particularly vulnerable to cryoinjury during the freezing process, which significantly compromises their post-thaw quality compared to spermatozoa maintained at ambient temperature [1,2]. The current focal point of research in semen cryopreservation is the mitigation of spermatozoa damage during the freezing process and the enhancement of spermatozoa quality post thawing. Yolks play an indispensable role in semen cryopreservation by maintaining the integrity of cell membranes, reducing cold stress, and providing nutrients [3,4,5]. In recent years, researchers have incorporated soy lecithin and low-density lipoprotein (LDL) into diluents as substitutes for egg yolk, yielding promising outcomes. However, the complexity and high costs associated with this approach have precluded it from fully supplanting the advantages offered by egg yolks in semen cryopreservation [6,7]. However, the protein composition of egg yolks is complex, and they contain a large amount of lipids, leading to an increase in the viscosity of the diluent. It has also been found that untreated egg yolk diluent contains large particulate matter, which reduces sperm motility [8]. This perspective is supported by a study conducted by Wang et al. [9], which demonstrated that high-pressure homogenization of chicken egg yolk resulted in a significant reduction in particle diameter. Furthermore, the study found that the quality of porcine spermatozoa was markedly enhanced following the processes of freezing and thawing.

Centrifugation is a simple and efficient way to remove large particles from a solution, and the viscosity of yolk dilutions is reduced after centrifugation, while the active ingredients in the dilutions are retained [10,11,12]. However, there is limited research on the application of centrifuged yolk diluents in the cryopreservation of porcine semen. In the present study, chicken yolk cryodiluent was subjected to centrifugation for the purpose of porcine semen cryopreservation, and its quality after freezing and thawing was evaluated to provide a reference basis for the optimization of porcine semen cryopreservation systems.

## 2. Materials and Methods

### 2.1. Experimental Materials

Five healthy adult boars were selected for this study. Prior to the formal experiment, semen was collected from each boar 2–3 times per week for 6 weeks to ensure a consistent semen quality. The criteria for cryopreservation were as follows: The semen samples had to be transported to the laboratory within 30 min of collection. Sperm motility was subjectively evaluated under a microscope, with a motility rate ≥ 70% and an abnormality rate ≤ 15%. Only healthy ejaculates were used in the experiments. After the initial assessment, the semen samples were mixed to eliminate individual differences.

#### 2.1.1. Major Reagents

The sperm viability assay kit was purchased from Regen Biotechnology Co., Ltd.; Beijing; China. Modified Giemsa stain, MDA, T-AOC, and CAT assay kits were obtained from the Nanjing Jiancheng Bioengineering Institute. Hoechst 33342 staining solution was purchased from Jiangsu KeyGEN BioTECH Co., Ltd.; Nanjing; China. Meanwhile, 2x Color SYBR Green qPCR Master Mix (ROX2) was obtained from EZBioscience Biotechnology Co., Ltd.; Jiangsu; China. The Evo M-MLV reverse-transcription reagent premix was obtained from Accurate Biotechnology Co., Ltd.; Wuhan; China.

#### 2.1.2. Major Instruments and Equipment

The main instruments and equipment used included the following: refrigerated centrifuge (ROTINA 380R, Hettich, Kirchlengern, Germany), microplate reader (MULTISKAN GO, Thermo Scientific, Waltham, MA, USA), computer-assisted sperm analysis (ML-608JZ, Nanning Songjing Tianlun Bioengineering Co., Ltd., Nanning, China), nucleic acid and protein analyzer (NANODROP ONE, Thermo Scientific, USA), real-time PCR system (FQD-96A, Hangzhou Bioer Technology Co., Ltd., Hangzhou, China), Fluorescence microscope (BX63, Olympus, Tokyo, Japan), and phase-contrast microscope (AE2000, Motic China Group Co., Ltd., Xiamen, China).

### 2.2. Extender Preparation

The method for preparing the cryopreservation base extender followed the approach of Gou [13]. The basic cryopreservation medium was Tris–citric–glucose (TCG). The TCG solution (100 mL) contained 2.42 g Tris, 1.48 g citric, 1.10 g glucose, and 100,000 IU penicillin–streptomycin solutions. The control group (CG)’s cryopreservation extender was prepared by mixing 20% egg yolk, 3% glycerol, and 77% base extender, which were homogenized using a magnetic stirrer. The experimental group (EG)’s cryopreservation extender was prepared by centrifuging the CG extender at 6000 rcf/min for 30 min at 4 °C, then collecting and storing the supernatant at 4 °C for later use.

### 2.3. Semen Processing

The qualified semen is thoroughly mixed, aliquoted into centrifuge tubes, and then centrifuged at 1500 r/min for 3 min to remove seminal plasma. The semen was resuspended in either the GE or GC cryopreservation diluent. The sperm pellet was resuspended in the cryopreservation extender.

### 2.4. Semen Freezing and Thawing

The semen was mixed evenly using a pipette and aliquoted into 0.5 mL cryopreservation straws, which were manually sealed with polyethylene powder. These were then exposed to vapor-phase nitrogen at a distance of 3 cm above the liquid nitrogen surface for a duration of 10 min prior to immersion in liquid nitrogen for storage. For thawing, the cryopreservation straws were removed from the liquid nitrogen and warmed in a 37 °C water bath for 50 s. The ends of the straws were cut, and the thawed semen was collected into 2 mL centrifuge tubes for further analysis.

### 2.5. Sperm Quality Assessment

#### 2.5.1. Sperm Motility Parameters

We added 10 μL of the thawed semen sample to a 20-micron Leja specialized slide and then placed the slide on a 37 °C heating plate for 30 s. The sperm motility parameters were analyzed using the computer-assisted sperm analysis (CASA) system, with nine random fields of view selected for evaluation. The recorded parameters included sperm motility, sperm vitality, average path velocity (VAP), straight-line velocity (VSL), curvilinear velocity (VCL), straightness (STR), linearity (LIN), and wobble (WOB).The software settings used for the CASA system were those recommended by the manufacturer for the analysis of boar spermatozoa: frame required 45, frame rate 60 Hz, minimum cell contrast 46, minimum cell size 7 pixels, straightness threshold 45%, velocity average path (VAP, micrometers per second) threshold 45 μm/s, low VAP cut-off 20 μm/s, low straight-line velocity (VSL, micrometers per second) cut-off 5.0 μm/s, static intensity gates 0.50–2.50, static size gates 0.65–4.90, and static elongation 0–87.

#### 2.5.2. Acrosome Integrity

According to the method described by Brum [14], with slight modifications, the integrity of the acrosome was evaluated using Coomassie Brilliant Blue staining. The procedure was meticulously executed in accordance with the manufacturer’s instructions, and the spermatozoa were examined under a high-power microscope at 400× magnification. Sperm with intact acrosomes exhibited a smooth, oval head devoid of any perforations or fissures, and a clearly defined crescent-shaped ridge was observed. Based on these observations, 200 spermatozoa were counted in each group to calculate the acrosome integrity rate.

#### 2.5.3. Plasma Membrane Integrity

The integrity of the sperm plasma membrane was assessed using a hypo-osmotic solution. According to the kit instructions, 0.1 mL of thawed sperm was added to 1 mL of hypo-osmotic solution and incubated in a 37 °C water bath for 60 min. Sperm with a curled tail was considered membrane-intact. A total of 200 sperm were counted to calculate the membrane integrity rate.

#### 2.5.4. Oxidative Stress Markers

Oxidative stress markers, including malondialdehyde (MDA), total antioxidant capacity (T-AOC), and catalase (CAT), were measured using assay kits. Three samples were taken from each group, and the procedures were strictly followed according to the kit instructions. The absorbance was measured using a microplate reader, and the results were calculated using the formula provided in the kit.

#### 2.5.5. Sperm Apoptosis Rate

Sperm apoptosis was assessed using Hoechst 33342 staining solution. The procedure was performed according to the kit instructions, and at least 100 sperm per group were analyzed under a fluorescence microscope. Apoptotic sperm displayed enhanced membrane permeability, allowing more dye to enter compared to normal sperm. As a result, apoptotic sperm exhibited a higher fluorescence intensity, showing a more intense blue color.

#### 2.5.6. Total RNA Extraction from Sperm

Total RNA was extracted from thawed sperm samples in each group by adding an equal volume of Trizol reagent. The specific procedure was as follows: We added 500 μL of lysis buffer to an RNase-free tip, rinsed 3–5 times, and then transferred the sample to an RNase-free centrifuge tube. We incubated the sample at room temperature for 10 min, added 100 μL of chloroform, vortexed vigorously for 15 s, incubated at room temperature for 3 min, and centrifuged at 12,000 rcf/min at 4 °C for 10 min (at which point the sample separated into three layers, with RNA in the upper aqueous phase). Then, we carefully transferred the upper aqueous phase (avoiding taking any of the middle layer’s material) to a clean RNase-free centrifuge tube, added an equal volume of isopropanol, mixed well, and incubated at room temperature for 20 min. We centrifuged the sample at 12,000 rcf/min at 4 °C for 10 min and discarded the supernatant. We then added 500 μL of pre-chilled 75% ethanol (prepared with DEPC water) to wash the pellet, centrifuged at 12,000 rcf/min at 4 °C for 3 min, and discarded the supernatant. We air-dried the pellet at room temperature for 10 min. We added 35 μL of RNase-free ddH_2_O and fully dissolved the RNA. We use NanoDrop to measure the total RNA concentration. RNA extraction was performed following the recommended protocol. The OD value (OD260/280) was measured to ensure the purity of the RNA, with acceptable values between 1.8 and 2.0. The RNA was maintained at −80 °C for future purposes.

#### 2.5.7. Reverse Transcription and qRT-PCR

Using a reverse-transcription reagent kit, total RNA samples were converted into cDNA, which was then stored at −20 °C. GAPDH was used as the reference gene. The gene sequences of interest (TNF-a, Bcl-2, Caspase-9, Bax, and CAT) were obtained from the NCBI database. Primer 5.0 software was utilized to design the primers. The primer sequences are shown in Table 1. For the qRT-PCR, the reaction conditions included an initial denaturation at 95 °C for 5 min, then 40 cycles of 10 s denaturation at 95 °C, and 30 s annealing at 60 °C.

### 2.6. Statistical Analysis

The data were subjected to paired-sample *t*-test using SPSS 26.0 software, and the results were expressed as the mean ± standard deviation, with *p* < 0.05 indicating significant differences.

## 3. Results

### 3.1. Particle Size of Egg Yolk After Centrifugation

**Figure 1:**
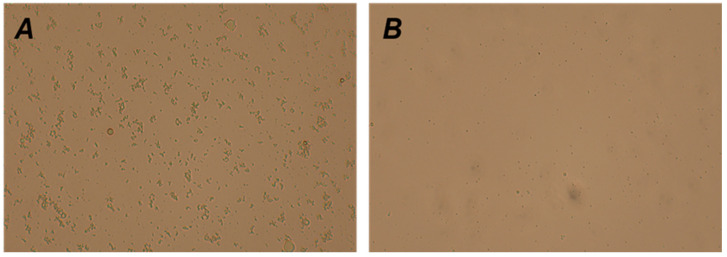
Microscopic structural analysis of the extender (200×): (**A**) image of the diluent without centrifugation and (**B**) image of the diluent released by centrifugation.

As shown in Figure 1, the non-centrifuged egg yolk extender contained densely packed and relatively large particles with an irregular distribution. After centrifugation, the number of particles was significantly reduced, and the particle size was noticeably smaller. Centrifugation effectively removed particulate matter from the egg yolk extender.

### 3.2. Effects of Centrifuged Egg Yolk on Post-Thaw Boar Sperm Motility Parameters

As shown in Table 2, compared with the CG, sperm motility and viability in the EG increased (*p* < 0.05). Additionally, VSL, VCL, and STR were significantly higher in the EG compared to the CG (*p* < 0.05); VAP and LIN also increased, although the differences were not statistically significant.

### 3.3. Effects of Centrifuged Egg Yolk on Post-Thaw Boar Sperm Morphology

**Figure 2:**
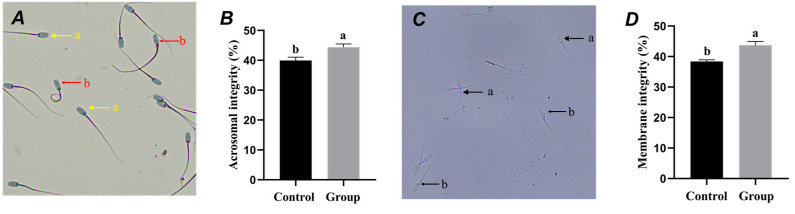
Effects of centrifuged egg yolk on post-thaw boar sperm morphology. (**A**) Acrosome morphology images: (**a**) sperm with intact acrosome; (**b**) sperm with incomplete acrosome; (**B**) Acrosome integrity test results (different lowercase letters indicate significant differences among groups, *p* < 0.05); (**C**) Plasma membrane morphology images: (**a**) sperm with intact plasma membrane; (**b**) sperm with incomplete plasma membrane; (**D**) Plasma membrane integrity test results (different lowercase letters indicate significant differences among groups, *p* < 0.05).

As shown in Figure 2A, the results of the Coomassie Brilliant Blue staining method indicate that acrosome-intact sperm exhibited a clear acrosome region with a uniform staining pattern. Compared with the CG, the acrosome integrity rate in the EG was significantly higher. The results of the hypo-osmotic swelling test show that sperm with intact plasma membranes displayed a curved tail (Figure 2C). The plasma membrane integrity rate in the EG was significantly higher than that of the CG.

### 3.4. Effect of Centrifuged Egg Yolk on Antioxidant Enzyme Content in Post-Thaw Boar Sperm

**Figure 3:**
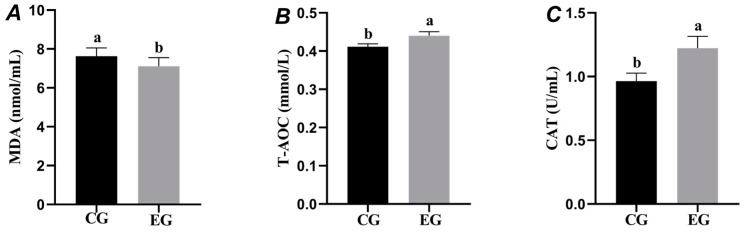
Effects of centrifuged egg yolk on post-thaw boar sperm oxidative stress markers. Different lowercase letters indicate significant differences among groups, *p* < 0.05.

As shown in Figure 3A, the MDA content in the EG was significantly lower than that in the CG (*p* < 0.05). Figure 3B,C show that the T-AOC and CAT levels in the EG were significantly higher compared to the CG (*p* < 0.05). 

### 3.5. Effects of Centrifuged Egg Yolk on Post-Thaw Boar Sperm Apoptosis Rate

**Figure 4:**
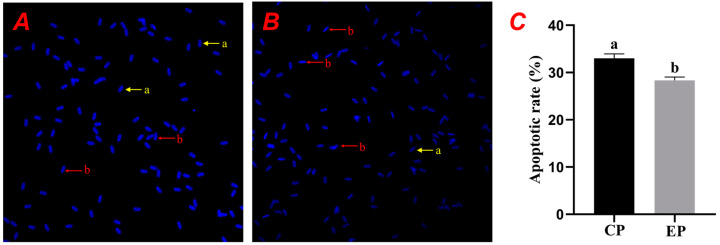
Effects of centrifuged egg yolk on post-thaw boar sperm apoptosis rate. (**A**,**B**) represent the staining results of sperm cells treated with Hoechst 33342, while (**C**) shows the sperm apoptosis rate (different lowercase letters indicate significant differences between groups, *p* < 0.05).

As shown in Figure 4A,B, the fluorescence intensity of sperm in the CG was higher than that in the EG. The increased membrane permeability in apoptotic cells allowed more dye to enter, resulting in stronger fluorescence, while normal cells exhibited weak fluorescence, and dead cells were impermeable to the dye. As shown in Figure 4C, the sperm apoptosis rate in the CG was significantly higher than in the EG (*p* < 0.05), consistent with the staining results.

### 3.6. Effects of Centrifuged Egg Yolk on Post-Thaw Boar Sperm Apoptosis-Related Gene Expression

**Figure 5:**
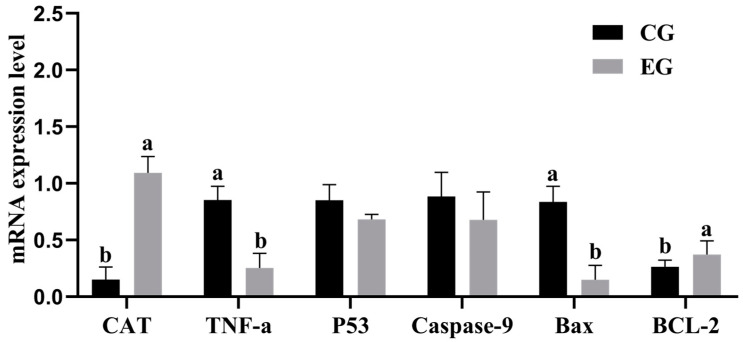
Effects of centrifuged egg yolk on post-thaw boar sperm apoptosis-related gene expression. (different lowercase letters indicate significant differences between groups, *p* < 0.05).

Figure 5 illustrates that the gene expression levels of TNF-a and Bax in the EG were significantly lower than those in the CG (*p* < 0.05). The expression levels of P53 and Caspase-9 were also lower in the EG compared to the CG, although the difference was not statistically significant (*p* > 0.05). Compared to the CG, the EG had significantly higher levels of CAT and Bcl-2 gene expression (*p* < 0.05).

## 4. Discussion

### 4.1. Effect of Centrifugal Treatment of Chicken Yolk Dilution on Particle Diameter

Based on the protective effect of egg yolk in semen extenders on sperm, the impact of different processing methods on the composition and function of egg yolk has garnered significant attention. In this study, centrifugation effectively removed particulate matter from the solution, in contrast to ultrasonication and high-pressure homogenization, which primarily facilitated the physical fragmentation of larger yolk particles into smaller constituents. Beatrice et al. [15] showed that, by centrifugation, the yolk could be separated into different fractions, mainly consisting of yolk plasma and yolk granules. Yolk plasma is mainly composed of 85% LDL and 15% globular glycoproteins [16]. Notably, LDL plays a crucial protective role in safeguarding sperm, mitigating cold-induced damage during the processes of sperm cryopreservation and subsequent thawing [17]. Currently, the cryoprotective properties of LDL on sperm have been substantiated across various livestock species [18,19]. At the same time, the widespread availability and established efficacy of centrifugation technologies in laboratory settings facilitate the potential for its extensive application.

### 4.2. Effect of Centrifugal Treatment of Chicken Egg Yolk on the Motility Parameters of Frozen and Thawed Porcine Spermatozoa

The results of this study showed that the granular matter in chicken yolk diluent after centrifugation was significantly less, and the motility parameters of sperm were improved. Oriza et al. [20] reported that an excess of yolk particles led to diminished sperm motility. However, the application of a cryoprotective solution containing 1% yolk significantly enhanced motility, whereas an increase in yolk concentration to 5% resulted in a decline in sperm motility. The application of centrifugation-treated diluent proved effective in minimizing sperm contact with yolk particles during motility, thereby reducing mechanical damage. Divar et al. [21] demonstrated that the quality of frozen–thawed spermatozoa improved when the diluent was cryopreserved in canine spermatozoa after a filtration treatment. The results of this experiment showed that sperm viability and activity were significantly increased in the centrifugation-treated group, and VSL, VCL, and STR were increased compared to the CG, with significant differences. The improvement of sperm motility parameters may be due to the reduction in large particulate matter in the diluent, the removal of harmful macromolecular matter by centrifugation, and the preservation of small molecular matter that has a protective effect on sperm. At the same time, the turbidity of the diluent and the presence of particulate matter can interfere with sperm detection, which is significantly improved by centrifugation.

### 4.3. Effect of Centrifugal Treatment of Chicken Yolk on the Morphology of Frozen and Thawed Porcine Spermatozoa

The yolk is abundant in LDL, lipids, and vitamins, which may offer protective benefits during the cryopreservation of sperm. The phospholipids present in the yolk have been shown to stabilize cell membranes and mitigate the physical damage to sperm induced by ice crystal formation during the freezing process [22]. The production of ice crystals is a primary factor contributing to cellular membrane damage during the cryopreservation of spermatozoa. Upon freezing, water crystallizes both intracellularly and extracellularly, potentially compromising membrane integrity and resulting in cellular demise. Some studies have reported that the presence of yolk granules may lead to the distribution of inhomogeneous ice crystals, which can exacerbate cell membrane damage [23]. The release of acrosomal enzymes and the fluidity of the plasma membrane together determine whether sperm can successfully penetrate the protective layer of the egg and bind to it, and the integrity of the acrosome and plasma membrane is crucial for sperm to be able to fertilize the egg properly [24]. The findings of this study demonstrated that, following the centrifugation of chicken yolk, there was a significant increase in both the acrosome integrity rate and the plasma membrane integrity rate in the EG. Yolk granules contain 70% of yolk high-density lipoprotein (HDL), a liquid lipid which may affect the cholesterol homeostasis of spermatozoa and, consequently, their physiological functions. Freezing and thawing diminish sperm motility fertility by disrupting the cholesterol balance in plasma organelle membranes [25]. During the freezing–thawing process, spermatozoa undergo oxidative stress, leading to an increase in intracellular reactive oxygen species (ROS) levels, which results in lipid peroxidation and subsequently impairs sperm function [26]. Natural antioxidants present in egg yolks, such as vitamin E (α-tocopherol), carotenoids, and glutathione, can effectively protect sperm from oxidative damage through multiple mechanisms, including scavenging ROS, inhibiting lipid peroxidation, and maintaining intracellular redox balance [27].

### 4.4. Effect of Centrifugal Treatment of Chicken Egg Yolk on Antioxidant Capacity of Frozen and Thawed Porcine Spermatozoa

Vitamins, lecithin, LDL, and carotenoids contained in egg yolk are able to scavenge oxidative free radicals and reduce the damage caused by oxidative stress to spermatozoa [28]. LDL also possesses antioxidant properties and is able to reduce the amount of free radicals generated during the freezing process and reduce spermatozoa damage caused by oxidative stress [29]. After centrifugation, the macromolecular substances in the yolk are effectively removed, while the lipids are largely preserved, primarily in the form of LDL [30]. The inclusion of LDL in the diluent mitigates cholesterol efflux and, due to its high antioxidant activity, effectively reduces the generation of reactive oxygen species during sperm cryopreservation. This process facilitates the formation of a functional CatSper channel, which is crucial for maintaining sperm motility, chemotaxis, and the acrosome’s reaction [31]. The findings of the current study demonstrated a significant reduction in MDA content, alongside a notable increase in T-AOC and CAT levels, in the test group relative to the CG. These results align with the outcomes reported in the aforementioned study.

### 4.5. Effect of Centrifugal Treatment of Chicken Egg Yolk on the Rate of Apoptosis in Frozen–Thawed Porcine Spermatozoa

The lipid-binding proteins contained in egg yolk interact with lipids in the sperm membrane to form a protective complex. This action not only prevents the loss of lipids but also stabilizes the cell membrane of the spermatozoa and thus reduces the transmission of pro-apoptotic signals [32]. The process of freezing and thawing results in the production of reactive oxygen species (ROS), oxygen free radicals which can cause damage to cellular DNA, proteins, and lipids, ultimately inducing apoptosis [33]. Egg yolks contain several antioxidant components that are capable of scavenging oxygen free radicals and reducing damage to spermatozoa from oxidative stress, which is one of the main factors leading to sperm apoptosis [34]. In this experiment, the apoptosis rate of freeze–thawed spermatozoa was detected using hoechst33342, and the results showed that the apoptosis rate of spermatozoa in the centrifuged chicken egg yolk dilution group was significantly lower than that of the CG. Similarly, in Wang’s study on the effect of high-pressure homogenization of egg yolk on the quality of frozen spermatozoa in pigs, the apoptosis rate of the CG decreased from 29.88% to 24.54%, and the DNA intact rate increased from 55.94% to 61.76% The difference between the two groups was significant [35]. During freezing, intracellular reactive oxygen species are produced, triggering oxidative stress responses which can damage the biomacromolecules inside the cells, including DNA, proteins, and lipids. Phosphatidylcholine, a type of phospholipid, is essential for cell membrane structure and helps stabilize sperm membranes, minimizing the risk of rupture during freezing and reducing apoptosis [36].

### 4.6. Effect of Centrifugal Treatment of Chicken Yolk on Apoptotic Gene Expression in Freeze–Thawed Porcine Spermatozoa

Apoptotic indicators, such as phosphatidylserine externalization and changes in mitochondrial membrane potential, are markedly elevated in spermatozoa following cryopreservation, suggesting the initiation of apoptosis [37]. The number of apoptotic spermatozoa during freezing and thawing is negatively correlated with sperm motility and viability [38]. Sperm apoptosis is influenced by a variety of factors, with cryoinjury due to low temperatures being a primary contributor to the decline in sperm quality [39]. Additionally, the elevation in ROS levels during freezing and thawing processes further exacerbates sperm apoptosis [40]. Key genes involved in apoptosis promotion include TNF-a, Caspase-9, and Bax, while Bcl-2 serves as an apoptosis inhibitor. Moreover, CAT functions as an antioxidant gene. Collectively, these genes play crucial roles in cellular processes [41]. In this study, TNF-a and Bax were significantly reduced, and the expression levels of Bcl-2 and CAT genes were significantly increased. The above results proved that the centrifugation of the chicken yolk dilution could effectively protect sperm cells, reduce the apoptosis rate, and improve the quality of frozen sperm. Wang et al. [35] investigated the effect of high-pressure homogenized egg yolk on the apoptosis of porcine spermatozoa, and the results showed that the TNF-a, Caspase-9, and Bax in the CG were significantly higher than those in the EG, and the expression level of the Bcl-2 gene was significantly reduced, which was consistent with the results of the present study. Evidence from some studies suggests that egg yolk can affect the expression of pro-apoptotic genes Bax and Bak, while upregulating the expression of anti-apoptotic genes Bcl-2 and Bcl-xl [42]. In this experiment, the yolk was centrifuged to improve the cryopreservation effect of porcine spermatozoa, a technique which can be further applied in future research on the addition of antioxidants, multi-species validation, and mechanism to enhance its practical application value in semen cryopreservation.

## 5. Conclusions

The results of this study showed that the particulate matter in the yolk was effectively removed after centrifugation, and the centrifugation-treated chicken yolk cryodilution inhibited oxidative damage during sperm freezing, improved sperm motility parameters, acrosomes, and plasma membranes, and reduced sperm apoptosis and the expression of apoptotic genes.

## Figures and Tables

**Table 1:** Primers of qRT-PCR.

Primer ID	Primer Sequence (5′–3′)	Product Length
GAPDH-F	TTCCACGGCACAGTCAAGGC	150 bp
GAPDH-R	CATGGTCGTGAAGACACCAG	
CAT-F	TGCCCATACTTCCCGTCC	172 bp
CAT-R	GGTCCAGGTTACCGTCAG	
TNF-a-F	ATTCAGGGATGTGTGGCCTG	120 bp
TNF-a-R	CCAGATGTCCCAGGTTGCAT	
P53-F	GAACAGsCTTTGAGGTGCGTG	175 bp
P53-R	GCCATCCAGTGGCTTCTTCT	
Caspase-9-F	AACTTCTGCCATGAGTCGGG	142 bp
Caspase-9-R	CCAAAGCCTGGACCATTTGC	
Bax-F	GCCGAAATGTTTGCTGACGG	146 bp
Bax-R	CGAAGGAAGTCCAGCGTCCA	
Bcl-2-F	GGCAACCCATCCTGGCACCT	134 bp
Bcl-2-R	AACTCATCGCCCGCCTCCCT	

**Table 2:** Effect of centrifugal treatment of yolk on sperm motility parameters after freezing and thawing.

Mobility Parameter	CG	EG	*p*-Value
Sperm viability, %	37.51 ± 0.91 ^b^	42.17 ± 1.46 ^a^	0.00
Progressive motility, %	30.09 ± 2.00 ^b^	34.46 ± 1.40 ^a^	0.00
VSL, μm/s	22.01 ± 1.59 ^b^	27.21 ± 1.48 ^a^	0.00
VCL, μm/s	44.72 ± 2.12 ^b^	50.34 ± 3.14 ^a^	0.00
VAP, μm/s	36.92 ± 3.60	39.35 ± 2.43	0.11
LIN, %	53.90 ± 3.73	54.24 ± 4.63	0.86
STR, %	59.91 ± 4.64 ^b^	69.34 ± 5.37 ^a^	0.00
WOB, %	82.44 ± 5.27	78.33 ± 5.28	0.11

VSL = straight-line velocity; VCL = curvilinear velocity; VAP = average path velocity; LIN = linearity; STR = straightness; and WOB = wobble. Columns within rows separated by differing letters are different (*p* < 0.05).

## Data Availability

No data were deposited in an official repository. All data generated during this study are available from the corresponding author upon request.
